# Objectively Measured Total Sedentary Time and Pattern of Sedentary Accumulation in Older Adults: Associations With Incident Cardiovascular Disease and All-Cause Mortality

**DOI:** 10.1093/gerona/glac023

**Published:** 2022-01-30

**Authors:** Manasa Shanta Yerramalla, Vincent T van Hees, Mathilde Chen, Aurore Fayosse, Sebastien F M Chastin, Séverine Sabia

**Affiliations:** 1 Université de Paris, INSERM U1153, Epidemiology of Ageing and Neurodegenerative Diseases, Paris,France; 2 Accelting, Almere, The Netherlands; 3 School of Health and Life Sciences, Glasgow Caledonian University, Glasgow, UK; 4 Department of Movement and Sports Sciences, Ghent University, Ghent, Belgium; 5 Department of Epidemiology and Public Health, University College London, London, UK

**Keywords:** Accelerometer, Breaks in sedentary behavior, Moderate-to-vigorous physical activity, Prospective

## Abstract

**Background:**

We examined associations of total duration and pattern of accumulation of objectively measured sedentary behavior (SB) with incident cardiovascular disease (CVD) and all-cause mortality among older adults.

**Methods:**

Total sedentary time and 8 sedentary accumulation pattern metrics were extracted from accelerometer data of 3 991 Whitehall II study participants aged 60–83 years in 2012–2013. Incident CVD and all-cause mortality were ascertained up to March 2019.

**Results:**

Two hundred and ninety-nine CVD cases and 260 deaths were recorded over a mean (standard deviation [*SD*]) follow-up of 6.2 (1.3) and 6.4 (0.8) years, respectively. Adjusting for sociodemographic and behavioral factors, 1-*SD* (100.2 minutes) increase in total sedentary time was associated with 20% higher CVD risk (hazard ratio [95% confidence interval]: 1.20 [1.05–1.37]). More fragmented SB was associated with reduced CVD risk (eg, 0.86 [0.76–0.97] for 1-*SD* [6.2] increase in breaks per sedentary hour). Associations were not evident once health-related factors and moderate-to-vigorous physical activity (MVPA) were considered. For all-cause mortality, associations with more fragmented SB (eg, 0.73 [0.59–0.91] for breaks per sedentary hour) were found only among the youngest older group (<74 years; *p* for interaction with age < .01) independently from all covariates.

**Conclusions:**

In this study, no associations of total sedentary time and sedentary accumulation patterns with incident CVD and all-cause mortality were found in the total sample once MVPA was considered. Our findings of reduced mortality risk with less total and more fragmented SB independent from MVPA among individuals <74 years need to be replicated to support the recent recommendations to reduce and fragment SB.

Sedentary behavior (SB) such as sitting is increasingly recognized as a risk factor for all-cause mortality (1) and cardiovascular disease (CVD) (2,3), and the extent to which its impact depends on the level of moderate-to-vigorous physical activity (MVPA) is raising research interest (4). It is suggested that not just the total duration being sedentary but also the manner in which it accumulates throughout the day (eg, in few long bouts or in several shorter bouts) might be important for health outcomes (5,6).

Experimental studies have reported that interrupting sedentary time with physical activity (PA) has acute benefits on controlling postprandial glucose and insulin levels (7,8). Such studies have shown that short PA breaks were slightly more effective for glycemic control than a continuous PA bout of a similar level of energy expenditure (8). Breaks in prolonged sitting have been shown to improve a wide range of cardiovascular parameters, especially blood pressure and vascular function (9). Taken together, this has led to recent PA guideline to incorporate specific recommendation of limiting and frequently interrupting time in SB (10,11), although evidence for these recommendations remains limited (11,12). However, over the last century technological advances have been accompanied with a large increase in the prevalence of SB (13,14). Identifying specific SB features, such as total duration or bout length (5,15), detrimental for health is thus necessary to inform future tailored interventions to tackle the impact of SB on health. This is particularly important for older adults who spend almost 80% of their time being sedentary (16).

To date few prospective studies have investigated the pattern of sedentary accumulation, with inconsistent findings for both incident CVD (17,18) and all-cause mortality (19,20). Only 2 studies on CVD risk that were sex-specific focused exclusively on older adults (17,18). Additionally, baring a single study among older women (18), the rest emphasized on either sedentary breaks or length of sedentary bouts as accumulation pattern measures. However, concept such as breaks has been described as crude measure to quantify accrual patterns, limited by its dependence on accelerometer wear time and inability to provide precise information on nature of breaks in terms of length or intensity (7,15). Use of measures that capture distribution of sedentary bout length and are sensitive to changes in SB has recently been recommended (21,22).

This study aimed to assess the association of objectively measured total sedentary time and the pattern of its accumulation with incident CVD and all-cause mortality among older adults. We also examined whether or not the associations were independent of MVPA. In the absence of a gold standard measure of sedentary accumulation patterns throughout the day, we used a comprehensive approach by investigating the association using 8 measures of SB accumulation patterns.

## Method

### Study Population

The Whitehall II study is a prospective cohort established in 1985–1988 among 10 308 London-based civil servants (67% males) aged 35–55 years (23). Since the inception of the study, sociodemographic, behavioral, and health-related factors have been assessed using questionnaires and clinical examinations. Follow-up assessments have taken place approximately every 4–5 years, with the latest wave completed in 2015–2016. Participants provided written informed consent. Research ethics approval was obtained from the University College London ethics committee (reference number 85/0938), renewed at each contact.

### Total Sedentary Time and Sedentary Accumulation Pattern

The accelerometer substudy was undertaken during the 2012–2013 wave of data collection for participants seen at the London clinic and for those living in the South-Eastern regions of England who underwent clinical examination at home. Participants were asked to wear a triaxial accelerometer (GENEActiv Original; Activinsights Ltd, Kimbolton, United Kingdom) on their nondominant wrist during 9 consecutive days over 24 hours. Data sampled at 85.7 Hz, with acceleration expressed relative to gravity (1*g* ≈ 9.81 m/s^2^), were processed in R software using GGIR package (24) version 2.3-3 (https://rdrr.io/cran/GGIR/). Euclidean Norm of raw accelerations Minus One with negative numbers rounded to zero was calculated (25). Sleep periods were then detected using a validated algorithm guided by sleep log (26). Data from the first waking up (Day 2) to waking up on the day before the last day (Day 8) were used, corresponding to 7 full days. Waking period was defined as the period between waking and onset of sleep. Participants were included for analysis if they had daily wear time ≥2/3 of waking hours, for at least 2 weekdays and 2 weekend days (27). Nonwear period among valid days was corrected based on a previously reported algorithm (25).

**Figure 1.**
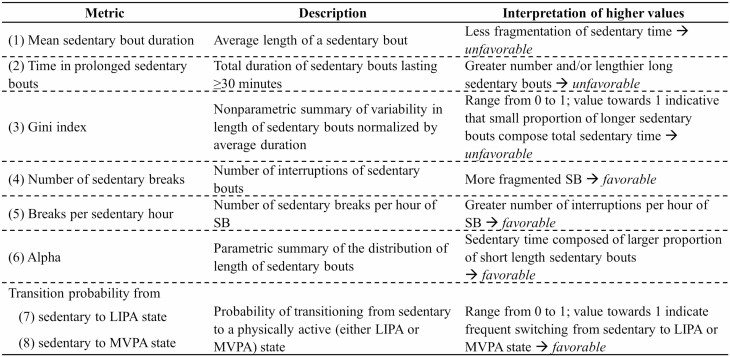
Description of metrics of sedentary accumulation pattern. *Notes*: LIPA = light intensity physical activity; MVPA = moderate-to-vigorous physical activity; SB = sedentary behavior.

Wrist-worn accelerometers have been reported to accurately classify movement behaviors based on metabolic intensity (28). In absence of gold standard cut points to classify movement behaviors in older adults, we used cut points based on a study wherein adult participants undertook 10 activities in laboratory in order to mimic free-living posture/behaviors with the aim to elicit average accelerations that were similar to those observed in a free-living situation (29). These cut points were in agreement with a recent study among older adults which derived cut points using oxygen consumption when performing 9 laboratory-based activities of daily living and showed good classification accuracy (30). Based on these studies, movement behavior during waking period was classified as SB when average acceleration over a 60-second epoch was <40 milligravity (m*g*), 40–99 m*g* for light intensity physical activity (LIPA), and ≥100 m*g* for MVPA (29,31). Sedentary accumulation pattern was measured using 8 metrics: mean sedentary bout duration (5), time in prolonged sedentary bouts, Gini index (32,33), number of sedentary breaks (32,33), breaks per sedentary hour (34,35), Alpha (32), and transition probability from sedentary to LIPA or MVPA states (Figure 1; see Supplementary Methods for description) (33).

Metrics (total time and accumulation pattern) were calculated for each day and averaged over 7 days. For those with <7 valid days (*N* = 95 [2.4%] participants), a weighted average was computed using data on weekend and weekdays (27). Test–retest analysis conducted among 79 participants who wore the accelerometer for 7 days on average 26.5 (standard deviation [*SD*] = 4.6) days after the first measure suggests a good reliability of all the measures (correlations range: 0.62–0.82).

### Ascertainment of CVD and All-Cause Mortality

CVD and mortality cases were ascertained by linkage to national registers up to the March 31, 2019 using the unique National Health Service identification number. *CVD* event was defined as occurrence of first fatal or nonfatal coronary heart disease (CHD), stroke, or heart failure. Nonfatal events were traced from the Hospital Episode Statistics (HES) database based on the International Classification of Diseases (ICD) codes for CHD (ICD-10 codes I20–25), stroke (ICD-10 codes I60–I64), and heart failure (ICD-10 code I50). CHD and stroke cases were also determined using Whitehall II study-specific 12-lead resting electrocardiogram recording and MONICA-Augsburg stroke questionnaire, respectively. Further details of validation of CVD cases are provided in a separate publication (36). CVD fatal events were drawn from the Office for National Statistics Mortality Register. *Death* from any cause was available from the UK Office for National Statistics Mortality Register.

### Ascertainment of Covariates

Covariates were assessed using questionnaire or during the clinical examination at 2012–2013 wave, as well as data from electronic health records including HES and the Mental Health Services Data Set. Sociodemographic variables consisted of sex, ethnicity (White, non-White), marital status (married/cohabitating, divorced/widowed/single), education (≤primary school, lower secondary, higher secondary school, university, higher degree; treated as continuous variable), last known occupational position (administrative, professional/executive, clerical/support). Behavioral factors included alcohol consumption (0, 1–14, >14 units per week), smoking status (current and recent ex-[less than 5 years] smokers, long-term ex-smokers, never smokers), fruits and vegetables consumption (<once daily, once daily, >once daily). Health-related factors consisted of prevalent diabetes (fasting glucose ≥ 7.0 mmol/L or self-reported doctor-diagnosed diabetes or use of diabetes medication or hospitalizations ascertained through record linkage to the HES [ICD-9 codes 250 or ICD-10 code E11]), body mass index (categorized as <24.9, 25–29.9, and ≥30 kg/m^2^), hypertension (systolic/diastolic blood pressure ≥ 140/90 mmHg or use of antihypertensive drugs), hyperlipidemia (low-density lipoproteins > 4.1 mmol/L or use of lipid-lowering drugs) assessed at the clinical examination, and morbidity index. For analysis on incident CVD, the morbidity index was calculated as the count of the following chronic conditions: cancer, arthritis, chronic obstructive pulmonary disease, depression, Parkinson disease, and dementia. For all-cause mortality the index additionally included CHD, stroke, and heart failure as chronic ailments.

### Statistical Analysis

For analysis on incident CVD, participants were censored at date of CVD, non-CVD related death to account for competing risks, or March 31, 2019 (end of follow-up), whichever came first. For all-cause mortality, censoring date was either date of death or end of follow-up (March 31, 2019), whichever came first. Four models were constructed. First model was adjusted for sociodemographic variables and total day duration (between awaking and sleep onset). Then, additionally adjusted for behavioral factors, followed by further adjustment for health-related factors. The final model included MVPA recommendation (<150 vs ≥150 minutes per week).

Potential nonlinear associations of total sedentary time and sedentary accumulation pattern metrics at 2012–2013 wave with incident CVD and all-cause mortality risk were tested using likelihood ratio test comparing fully adjusted Cox regression models with only linear term against models with cubic spline terms (37). When associations were deemed linear, exposures were treated as continuous variables in analyses. For ease of interpretability and comparability, exposures were standardized (mean = 0, *SD* = 1) using mean and *SD* from the largest analytical sample, one with mortality as outcome. All analyses were conducted using Cox regression with age as timescale. Proportionality assumption was verified using Schoenfeld’s test.

Owing to substantial correlations between total sedentary time and sedentary accumulation pattern metrics, they could not be mutually adjusted. Alternatively, we tested the interaction between total sedentary time (categorized using median split) and each sedentary accumulation pattern metric. We also tested interactions with age (continuous), sex, obesity (<30 and ≥30 kg/m^2^), and morbidity (0 and ≥1 prevalent chronic ailment). When interactions were found, analyses were repeated separately in each group (for age, groups were split as <74 and ≥74 years to allow enough cases in each group). All analyses were undertaken using Stata statistical software version 15 (StataCorp, College Station, TX) and R version 3.6.3 (http://www.r-project.org) with a 2-sided *p* < .05 considered statistically significant.

### Sensitivity Analysis

Three sets of sensitivity analyses were conducted. First, to examine potential for reverse causation, main analysis was repeated by excluding CVD events and death occurring within first 2 years of follow-up for incident CVD and all-cause mortality outcomes, respectively. Second, the stratified analysis on age for all-cause mortality was repeated using an alternative age cut point based on median age split. Third, the main analyses were repeated by adjusting for MVPA as a continuous instead of as a dichotomous variable.

## Results

### Participant Characteristics

Among the 6 308 participants in the 2012–2013 wave, 4 880 were invited to participate in the accelerometer substudy, with 4 492 agreeing and 4 008 returning the devices successfully with valid data (Supplementary Figure 1). Excluding those with preexisting CVD (for incident CVD outcome) or missing covariates led to an analytical sample of 3 321 participants for analysis on incident CVD and 3 991 for all-cause mortality. Compared with participants invited to the accelerometer substudy (*n* = 4 880) and subsequently included (*n* = 3 991) in the analyses, participants not included (*n* = 889) were on average younger (excluded vs included participants: 68.9 vs 69.4 years, *p* = .03), more likely to be women (33.5% vs 25.8%, *p* < .001), non-White (10.5% vs 7.4%, *p* < .01), and had higher education level (36.6% vs 31.0%, *p* < .01) (Supplementary Table 1). During a mean follow-up of 6.2 (*SD* = 1.3) years, there were a total of 299 incident CVD events (CHD [62.9%], stroke [17.7%], and heart failure [19.4%]). A total of 260 all-cause deaths were recorded over a mean follow-up of 6.4 (*SD* = 0.8) years.

Participants with incident CVD events were more likely to be older, men, non-White, less educated, smokers, and have worse cardiometabolic profile compared to those who did not develop CVD over the follow-up (Table 1). Those who died were more likely to be older, married/cohabitating, less educated, have poorer diet, worse cardiometabolic profile, and more comorbidities than surviving participants (Table 1). Participants with incident CVD or all-cause death were likely to spend more time in SB, accumulate sedentary time in longer bouts and with fewer interruptions, and were less likely to switch from sedentary to LIPA and MVPA states compared to those without the event of interest (Table 1). The correlations of total sedentary time with the 8 sedentary accumulation metrics ranged from 0.45 (Gini index) to 0.88 (time in prolonged [≥30 minutes] sedentary bouts) in absolute term (Supplementary Table 2). Time in MVPA was moderately correlated with most variables (*r* = 0.27–0.67 in absolute term).

**Table 1.** Baseline Characteristics of Study Participants

	Incident CVD (*N* = 3 321)			All-Cause Mortality (*N* = 3 991)		
Characteristics	No	Yes	*p* Value	No	Yes	*p* Value
*N* (row %)	3 022 (91.0)	299 (9.0)		3 731 (93.5)	260 (6.5)	
Age (years), *M* (*SD*)	68.6 (5.5)	71.5 (5.9)	<.001	69.1 (6.0)	73.7 (5.4)	<.001
Women	830 (27.5)	55 (18.4)	.001	967 (25.9)	63 (24.2)	.55
Non-White	173 (5.7)	33 (11.0)	<.001	272 (7.3)	23 (8.9)	.35
Married/cohabitating	2 264 (74.9)	226 (75.6)	.80	2 802 (75.1)	179 (68.9)	.03
University or higher degree	995 (32.9)	76 (25.4)	.01	1 175 (31.5)	63 (24.2)	.01
Low occupational position	1 495 (49.5)	155 (51.8)	.42	1 885 (50.5)	146 (56.2)	.08
Recent-ex/current smokers	152 (5.0)	23 (7.7)	.05	205 (5.5)	16 (6.2)	.65
>14 units of alcohol per week	716 (23.7)	72 (24.1)	.88	875 (23.5)	50 (19.2)	.12
Daily intake of fruits and vegetable	2 424 (80.2)	227 (75.9)	.08	2 972 (79.7)	193 (74.2)	.04
BMI ≥ 30 kg/m^2^	496 (16.4)	62 (20.7)	.06	678 (18.2)	45 (17.3)	.73
Hypertension*	1 347 (44.6)	183 (61.2)	<.001	1 899 (50.9)	167 (64.2)	<.001
Hyperlipidemia†	1 365 (45.2)	150 (50.2)	.10	1 885 (50.5)	136 (52.3)	.58
Diabetes	311 (10.3)	58 (19.4)	<.001	461 (12.4)	53 (20.4)	<.001
Morbidity index,‡*M* (*SD*)	0.33 (0.6)	0.36 (0.6)	.36	0.52 (0.7)	0.89 (1.0)	<.001
Following recommendations of 150 min/day of MVPA	2 592 (85.8)	216 (72.2)	<.001	3 116 (83.5)	154 (59.2)	<.001
Sedentary time variables, *M* (*SD*)						
Daily sedentary time, min/day	709.5 (98.2)	741.3 (110.0)	<.001	714.9 (99.1)	760.5 (105.6)	<.001
Mean sedentary bout duration	11.0 (5.2)	12.9 (9.5)	<.001	11.3 (5.8)	13.9 (8.8)	<.001
Time in prolonged (≥30 min) sedentary bouts, min/day	372.5 (138.1)	417.5 (162.4)	<.001	380.1 (140.5)	452.3 (164.0)	<.001
Gini index	0.67 (0.04)	0.68 (0.03)	.004	0.68 (0.04)	0.69 (0.04)	<.001
Number of sedentary breaks	71.7 (15.4)	68.8 (18.1)	.002	71.2 (15.7)	65.8 (18.4)	<.001
Breaks per sedentary hour	6.4 (1.9)	5.9 (2.1)	<.001	6.3 (1.9)	5.5 (2.0)	<.001
Alpha	1.76 (0.13)	1.73 (0.14)	<.001	1.76 (0.13)	1.71 (0.14)	<.001
Transition probability (%) from						
sedentary to LIPA state	9.9 (3.0)	8.8 (3.3)	.001	10.0 (3.0)	9.4 (3.3)	<.001
sedentary to MVPA state	0.55 (0.48)	0.32 (0.36)	<.001	0.57 (0.49)	0.43 (0.41)	<.001

*Notes*: BMI = body mass index; CVD = cardiovascular disease; LIPA = light intensity physical activity; *M* = mean; MVPA = moderate-to-vigorous physical activity; *SD* = standard deviation. Values are *N* (col %) unless otherwise stated.

*Systolic/diastolic blood pressure ≥ 140/90 mmHg or use of antihypertensive drugs.

^†^Low-density lipoprotein ≥ 4.1 mmol/L or use of lipid-lowering drugs.

^‡^Number of chronic conditions among: cancer, arthritis, chronic obstructive pulmonary disease, depression, Parkinson disease, and dementia for incident CVD. Addition of coronary heart disease, stroke, and heart failure for all-cause mortality.

There was no evidence of a nonlinear relationship of total sedentary time and sedentary accumulation metrics with incident CVD (*p* nonlinearity range: 0.06–0.72) and all-cause mortality (*p* nonlinearity range: 0.14–0.94), so all variables were examined as continuous variables in the models. All SB measures were standardized so that 1 *SD* represents 100.2 minutes for total sedentary time, 6.1 minutes for mean sedentary bout duration, 143.2 minutes for time in prolonged (≥30 minutes) sedentary bouts, 0.036 for Gini index, 16.0 for number of sedentary breaks, 6.2 for breaks per sedentary hour, 0.127 for Alpha, and 3.1% and 0.5% for transition probability from sedentary to LIPA and MVPA states, respectively.

### Association of Sedentary Time and Its Accumulation Pattern With Incident CVD

Table 2 shows the associations of total sedentary time and sedentary accumulation pattern metrics with incident CVD. In analysis adjusted for sociodemographic factors, 1-*SD* higher total sedentary time was associated with higher risk of incident CVD (hazard ratio [HR] 1.20, 95% confidence interval 1.06–1.37). All accumulation measures, except Gini index were significantly associated with CVD risk. A 1-*SD* increase in mean sedentary bout duration (HR 1.18, 1.08–1.30) and in prolonged sedentary bout duration (HR 1.18, 1.06–1.33) was associated with 18% increase in CVD risk while 1-*SD* increase in transition probability from sedentary to MVPA state (HR 0.81, 0.70–0.94) was associated with the largest decrease in CVD risk. No changes were observed in risk estimates when adjusting for behavioral factors. After additional adjustment for health-related factors, the association remained only for mean sedentary bout duration (HR 1.14, 1.03–1.26) and was no longer significant on further adjustment for MVPA (HR 1.09, 0.98–1.23). In models adjusted for MVPA but not health-related factors, associations were no more evident either, except for mean sedentary bout duration which had borderline significance (HR 1.12, 1.00–1.24, *p* = .045; Supplementary Table 3). As a comparison, the HR for meeting the MVPA recommendation of 150 minutes per week was 0.69 (0.52–0.92, *p* = .01) in a model adjusted for sociodemographic, behavioral, and health-related factors (Supplementary Table 4). Associations of sedentary accumulation metrics with CVD risk did not vary by total sedentary time (*p* interaction: .07–.57). There was no evidence that age, sex, obesity, or morbidity status modified the association of total sedentary time and metrics of sedentary accumulation pattern with incident CVD (all *p* interaction > .07).

**Table 2.** Associations of Total Sedentary Time and Sedentary Accumulation Patterns With Incident CVD (*N* total = 3 321, *N* events = 299, mean follow-up [*SD*] = 6.2 [1.3] years)

	HR (95% CI)			
	Adjusted for Sociodemographic Factors*	Additionally Adjusted for Behavioral Factors†	Additionally Adjusted for Health-Related Factors‡	Additionally Adjusted for MVPA§
Total sedentary time	1.20 (1.06–1.37)	1.20 (1.05–1.37)	1.11 (0.97–1.27)	1.02 (0.88–1.19)
Sedentary accumulation pattern metrics^‖^				
(1) Mean sedentary bout duration	1.18 (1.08–1.30)	1.19 (1.08–1.30)	1.14 (1.03–1.26)	1.09 (0.98–1.23)
(2) Time in prolonged (≥30 min) sedentary bouts	1.18 (1.06–1.33)	1.19 (1.06–1.33)	1.11 (0.99–1.26)	1.05 (0.92–1.20)
(3) Gini index	1.06 (0.94–1.19)	1.07 (0.95–1.21)	1.03 (0.91–1.16)	0.99 (0.88–1.12)
(4) Number of sedentary breaks	0.87 (0.77–0.97)	0.86 (0.77–0.97)	0.91 (0.81–1.02)	0.94 (0.84–1.07)
(5) Breaks per sedentary hour	0.86 (0.76–0.97)	0.86 (0.76–0.97)	0.92 (0.81–1.04)	0.97 (0.85–1.10)
(6) Alpha	0.84 (0.75–0.95)	0.85 (0.75–0.95)	0.90 (0.79–1.01)	0.94 (0.82–1.07)
Transition probability from				
(7) sedentary to LIPA state	0.88 (0.78–0.99)	0.87 (0.78–0.99)	0.93 (0.82–1.05)	0.98 (0.86–1.11)
(8) sedentary to MVPA state	0.81 (0.70–0.94)	0.82 (0.70–0.95)	0.87 (0.75–1.01)	0.92 (0.79–1.08)

*Notes*: BMI = body mass index; CI = confidence interval; CVD = cardiovascular disease; HR = hazard ratio; LIPA = light intensity physical activity; MVPA = moderate-to-vigorous physical activity; *SD* = standard deviation.

*Models adjusted for age (timescale), sex, ethnicity, education, occupation position, marital status, and total waking day duration.

^†^Models additionally adjusted for smoking status, alcohol consumption, and fruits and vegetables consumption.

^‡^Models additionally adjusted for prevalent diabetes, BMI, hypertension, hyperlipidemia, and morbidity index.

^§^Models additionally adjusted for MVPA recommendation.

^‖^Metrics are standardized based on sample mean and *SD* resulting in HRs corresponding to 1-*SD* higher value. For metrics 1–3, an increase of 1 *SD* corresponds to less favorable sedentary accumulation pattern. For metrics 4–8, an increase of 1 *SD* corresponds to more favorable sedentary accumulation pattern. 1 *SD* represents 100.2 minutes for total sedentary time, 6.1 minutes for mean sedentary bout duration, 143.2 minutes for time in prolonged (≥30 minutes) sedentary bouts, 0.036 for Gini index, 16.0 for number of sedentary breaks, 6.2 for breaks per sedentary hour, 0.127 for Alpha, and 3.1% and 0.5% for transition probability from sedentary to LIPA and MVPA states, respectively.

### Association of Sedentary Time and Its Accumulation Pattern With Mortality

The association of sedentary time and its accumulation pattern with all-cause mortality is shown in Table 3. In analysis adjusted for sociodemographic factors, a 1-*SD* increase in total sedentary time (HR 1.35, 1.17–1.56), mean sedentary bout duration (HR 1.10, 1.03–1.17), and prolonged sedentary bout duration (HR 1.27, 1.13–1.43) were associated with higher mortality risk. More fragmented SB pattern as shown by 1-*SD* increase in number of sedentary breaks (HR 0.83, 0.74–0.94), breaks per sedentary hour (HR 0.80, 0.70–0.91), Alpha (HR 0.81, 0.72–0.92), transition probability from sedentary to LIPA (HR 0.82, 0.72–0.93) and MVPA (HR 0.69, 0.57–0.84) states were associated with lower mortality risk. Additional adjustment for behavioral and health-related factors slightly attenuated the associations. On further adjustment for MVPA, none of the associations remained significant. Meeting the recommended MVPA duration was associated with a 41% reduction in mortality risk in the fully adjusted model (HR 0.59, 0.44–0.78; Supplementary Table 4). There was no evidence that the associations between sedentary accumulation metrics and mortality vary by sedentary time (*p* interaction: .42–.75).

**Table 3.** Associations of Total Sedentary Time and Sedentary Accumulation Patterns With All-Cause Mortality (*N* total = 3 991, *N* events = 260, mean follow-up [*SD*] = 6.4 [0.8] years)

	HR (95% CI)			
	Adjusted for Sociodemographic Factors*	Additionally Adjusted for Behavioral Factors†	Additionally Adjusted for Health-Related Factors‡	Additionally Adjusted for MVPA§
Total sedentary time	1.35 (1.17–1.56)	1.32 (1.15–1.53)	1.29 (1.11–1.49)	1.16 (0.98–1.38)
Sedentary accumulation pattern metrics‖				
(1) Mean sedentary bout duration	1.10 (1.03–1.17)	1.08 (1.01–1.15)	1.07 (1.00–1.15)	1.03 (0.95–1.11)
(2) Time in prolonged (≥30 min) sedentary bouts	1.27 (1.13–1.43)	1.25 (1.11–1.40)	1.22 (1.08–1.38)	1.12 (0.98–1.29)
(3) Gini index	1.13 (1.00–1.29)	1.13 (0.99–1.28)	1.11 (0.97–1.26)	1.06 (0.93–1.20)
(4) Number of sedentary breaks	0.83 (0.74–0.94)	0.85 (0.75–0.95)	0.86 (0.77–0.97)	0.92 (0.81–1.05)
(5) Breaks per sedentary hour	0.80 (0.70–0.91)	0.81 (0.72–0.93)	0.83 (0.73–0.95)	0.90 (0.78–1.04)
(6) Alpha	0.81 (0.72–0.92)	0.83 (0.73–0.94)	0.84 (0.75–0.96)	0.92 (0.80–1.05)
Transition probability from				
(7) sedentary to LIPA state	0.82 (0.72–0.93)	0.83 (0.73–0.95)	0.85 (0.75–0.97)	0.92 (0.80–1.06)
(8) sedentary to MVPA state	0.69 (0.57–0.84)	0.71 (0.58–0.86)	0.74 (0.61–0.90)	0.82 (0.67–1.01)

*Notes*: BMI = body mass index; CI = confidence interval; CVD = cardiovascular disease; HR = hazard ratio; LIPA = light intensity physical activity; MVPA = moderate-to-vigorous physical activity; *SD* = standard deviation.

*Models adjusted for age (timescale), sex, ethnicity, education, occupation position, marital status, and total waking day duration.

^†^Models additionally adjusted for smoking status, alcohol consumption, and fruits and vegetables consumption.

^‡^Models additionally adjusted for prevalent diabetes, BMI, hypertension, hyperlipidemia, and morbidity index.

^§^Models additionally adjusted for MVPA recommendation.

^‖^Metrics are standardized based on sample mean and *SD* resulting in HRs corresponding to 1-*SD* higher value. For metrics 1–3, an increase of 1 *SD* corresponds to less favorable sedentary accumulation pattern. For metrics 4–8, an increase of 1 *SD* corresponds to more favorable sedentary accumulation pattern. 1 *SD* represents 100.2 minutes for total sedentary time, 6.1 minutes for mean sedentary bout duration, 143.2 minutes for time in prolonged (≥30 minutes) sedentary bouts, 0.036 for Gini index, 16.0 for number of sedentary breaks, 6.2 for breaks per sedentary hour, 0.127 for Alpha, and 3.1% and 0.5% for transition probability from sedentary to LIPA and MVPA states, respectively.

**Figure 2.**
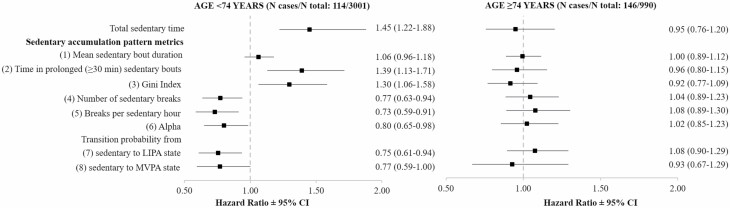
Associations of total sedentary time and sedentary accumulation patterns with all-cause mortality stratified by age. *Notes*: CI = confidence interval; LIPA = light intensity physical activity; MVPA = moderate-to-vigorous physical activity. Models adjusted for age (as timescale), sociodemographic, behavioral, health-related risk factors, and MVPA recommendation. Metrics are standardized based on sample mean and *SD* resulting in HRs corresponding to 1-*SD* higher value. For metrics 1–3, an increase of 1 *SD* corresponds to less favorable sedentary accumulation pattern. For metrics 4–8, an increase of 1 *SD* corresponds to more favorable sedentary accumulation pattern. 1 *SD* represents 100.2 minutes for total sedentary time, 6.1 minutes for mean sedentary bout duration, 143.2 minutes for time in prolonged (≥30 minutes) sedentary bouts, 0.036 for Gini index, 16.0 for number of sedentary breaks, 6.2 for breaks per sedentary hour, 0.127 for Alpha, and 3.1% and 0.5% for transition probability from sedentary to LIPA and MVPA states, respectively.

For all-cause mortality, a consistent interaction was observed between age and SB measures (*p* interaction: .001–.009, except for transition probability from sedentary to MVPA state where *p* = .18). In fully adjusted analyses stratified by age (Figure 2), total sedentary time and most SB accumulation metrics were significantly associated with all-cause mortality among those aged <74 years (*N* = 3 001, *N* death = 114; Supplementary Table 5), whereas there was no association among the oldest group (age ≥ 74 years, *N* = 990, *N* death = 146; Supplementary Table 6). While causes of death did not differ in both age groups, SB measures were on average better among younger group (Supplementary Table 7).

### Sensitivity Analysis

Excluding 88 CVD events within the first 2 years of follow-up (Supplementary Table 8) completely attenuated associations, including in model adjusted only for sociodemographic factors except for mean sedentary bout duration. Removing 45 all-cause mortality events within the first 2 years of follow-up either in the full population (Supplementary Table 9) or by age group (Supplementary Table 10) did not affect the findings. Using a median age split in analysis for all-cause mortality showed similar findings as in the main analyses with associations evident only in the youngest age group (<68.4 years) (Supplementary Figure 2). Adjusting for MVPA as a continuous instead of dichotomous variable in the final adjustment model did not change the findings (Supplementary Tables 11 and 12).

## Discussion

This prospective study based on objective measures of SB and PA in older adults with a mean follow-up of over 6 years presents 3 key findings. First, total sedentary time and all SB accumulation metrics, apart from the Gini index, were associated with incident CVD and death independently from sociodemographic and behavioral factors. Secondly, the observed association of total sedentary time and pattern of sedentary accumulation with incident CVD was explained by health-related factors and MVPA duration. Thirdly, among the youngest older adults, total sedentary time and most sedentary accumulation pattern measures remained associated with all-cause mortality even after accounting for health-related factors and MVPA, while no association was found irrespective of the metrics in the oldest old.

Studies based on self-reported measures (38,39) have found higher sedentary time to be associated with increased risk of CVD incidence, while conclusions are mixed for the limited number of studies using objective measures (17,18,40–42). A pooled analysis of 9 prospective studies, mean age of 54.4 years and median follow-up of 11 years, reported a nonlinear association of questionnaire assessed sedentary time with CVD incidence, with the increase in risk observed only at a duration greater than 10 hours/day, when adjusted for PA (38). In contrast using objectively assessed SB, the Objective Physical Activity and Cardiovascular Health (OPACH) study of older women found a linear dose–response relationship where each 1 additional hour of sedentary time was associated with 12% higher CVD risk (18) in model accounting for multiple risk factors and MVPA. In other studies the associations were attenuated on adjustment with higher intensity PA (17), health-related factors (41,42), or both (40), as also found in our study.

Till date 2 prospective studies have examined the association of sedentary accumulation pattern with incident CVD (17,18). A study based on older men did not find any association between sedentary breaks or bouts and CVD risk (17). In the OPACH study, longer mean sedentary bout duration, less breaks in sedentary time, and accumulating sedentary time in a prolonged manner were associated with higher CVD risk among older women (18). These associations persisted for mean sedentary bout duration and Alpha, on further adjustment for CVD risk factors and MVPA, albeit not mutually adjusted. In our study, adjustment for wide range of health-related factors including CVD risk markers attenuated the association apart for mean sedentary bout duration. This suggests a potential role of CVD risk markers in the association between SB metrics and CVD risk, which is in accordance with previous findings showing SB metrics associated with cardiometabolic risk factors in adults (43,44). In our case, this association was no more significant on either mutual or separate adjustment for MVPA. Findings might differ owing to metric utilized, adjustment level, and use of self-reported data for morbidity prevalence (18) unlike in our study which uses health-records linkage data.

A meta-analysis of 8 prospective studies found that longer accelerometer-assessed sedentary time was associated with increased all-cause mortality risk even after adjustment for MVPA (1). Only few observational studies have examined the associations between patterns of sedentary accumulation and all-cause mortality (19,20), but findings reported were inconsistent. In the present study, associations of total sedentary time and most SB accumulation pattern metrics with all-cause mortality differed as a function of age and were evident only among the youngest older adults even when accounting for a large set of confounders including MVPA. This could explain differences in findings between previous studies where a study based on adults with mean age of 63.5 years found higher number of breaks to be associated with lower mortality risk (19), while another study among older men with mean age of 78.4 years did not report any association using same measure (20). Another study based on the sample used in the former study (mean age = 63.5) found replacing prolonged sedentary bouts with shorter sedentary bouts not to be associated with reduced mortality risk, although it was the case for replacement with LIPA or MVPA (45). This is in line with our finding that increase in switching from sedentary to either LIPA or MVPA states is associated with reducing mortality risk in youngest older adults.

A potential explanation of the differential associations observed by age group is the better overall level of SB measures seen in the younger compared to the oldest group. Another possible reason could be due to the change in functional capacity over the life course, also termed as “fitness gap” (46). Among the oldest old population, as the capacity itself is lacking we would not expect to see association of SB with all-cause mortality.

Our study has several strengths. It is longitudinal, based on both men and women as compared to the earlier notable studies based only on men (20) or women (18), with exclusive focus on older adults. We controlled analyses for a wide range of factors such as CVD biological risk factors and diabetes prevalence which were ascertained using multiple objective sources including clinical examinations rather than being self-reported. Additionally, in the absence of a gold standard measure of accumulation of sedentary time, we used a large and comprehensive range of metrics as exposures on the same outcomes.

The limitations should also be noted. First, the Whitehall II study is an occupational cohort wherein participants are healthier than the general population, but it has been shown previously that the associations between cardiovascular risk factors, including PA, and CVD risk are similar to that in the general population (47). Second, we adjusted for a broad range of confounders, but a possibility of an unmeasured factor to further explain the association still exists.

## Conclusion

The 2018 United States of America Physical Activity guidelines and the 2020 World Health Organization guidelines on PA and SB concluded that there is insufficient evidence to indicate that sedentary breaks are important factors for incident CVD and all-cause mortality (11,12). In this study we examined associations of multiple sedentary accumulation pattern measures with both outcomes, given that different metrics might be indicative of distinct features of SB. Based on our findings, we reiterate the importance of MVPA for CVD prevention (42), as associations of total sedentary time and accumulation patterns with CVD risk were no more evident once MVPA was considered. In addition, there was evidence of higher all-cause mortality risk with increased total and less fragmented sedentary time independently from MVPA in the younger older adults. If these later findings are replicated in future studies, this would support the current Canadian recommendations (10) on limiting and interrupting long periods of sedentary time. Why such associations are not seen in the oldest group requires further investigation.

## Supplementary Material

glac023_suppl_Supplementary_Material

## References

[1] Ekelund U , Tarp J, Steene-Johannessen J, et al. Dose-response associations between accelerometry measured physical activity and sedentary time and all cause mortality: systematic review and harmonised meta-analysis. BMJ (Clinical Research Ed). 2019;366:l4570. doi:10.1136/bmj.l4570 10.1136/bmj.l4570 PMC6699591 31434697

[2] Biswas A , Oh PI, Faulkner GE, et al. Sedentary time and its association with risk for disease incidence, mortality, and hospitalization in adults: a systematic review and meta-analysis. Ann Intern Med. 2015;162(2):123–132. doi:10.7326/M14-1651 25599350 10.7326/M14-1651

[3] Patterson R , McNamara E, Tainio M, et al. Sedentary behaviour and risk of all-cause, cardiovascular and cancer mortality, and incident type 2 diabetes: a systematic review and dose response meta-analysis. Eur J Epidemiol. 2018;33(9):811–829. doi:10.1007/s10654-018-0380-1 29589226 10.1007/s10654-018-0380-1 PMC6133005

[4] Chastin S , McGregor D, Palarea-Albaladejo J, et al. Joint association between accelerometry-measured daily combination of time spent in physical activity, sedentary behaviour and sleep and all-cause mortality: a pooled analysis of six prospective cohorts using compositional analysis. Br J Sports Med. 2021;55(22):1277–1285. doi:10.1136/bjsports-2020-102345 34006506 10.1136/bjsports-2020-102345 PMC8543228

[5] Healy GN , Dunstan DW, Salmon J, et al. Breaks in sedentary time: beneficial associations with metabolic risk. Diabetes Care. 2008;31(4):661–666. doi:10.2337/dc07-2046 18252901 10.2337/dc07-2046

[6] Sardinha LB , Santos DA, Silva AM, Baptista F, Owen N. Breaking-up sedentary time is associated with physical function in older adults. J Gerontol A Biol Sci Med Sci. 2015;70(1):119–124. doi:10.1093/gerona/glu193 25324221 10.1093/gerona/glu193

[7] Chastin SF , Egerton T, Leask C, Stamatakis E. Meta-analysis of the relationship between breaks in sedentary behavior and cardiometabolic health. Obesity (Silver Spring, Md). 2015;23(9):1800–1810. doi:10.1002/oby.21180 10.1002/oby.21180 26308477

[8] Loh R , Stamatakis E, Folkerts D, Allgrove JE, Moir HJ. Effects of interrupting prolonged sitting with physical activity breaks on blood glucose, insulin and triacylglycerol measures: a systematic review and meta-analysis. Sports Medicine (Auckland, NZ). 2020;50(2):295–330. doi:10.1007/s40279-019-01183-w 10.1007/s40279-019-01183-w PMC6985064 31552570

[9] da Silva GO , Santini LB, Farah BQ, Germano-Soares AH, Correia MA, Ritti-Dias RM. Effects of breaking up prolonged sitting on cardiovascular parameters: a systematic review. Int J Sports Med. 2022;43(2):97–106. doi:10.1055/a-1502-6787 34535019 10.1055/a-1502-6787

[10] Ross R , Chaput JP, Giangregorio LM, et al. Canadian 24-hour movement guidelines for adults aged 18–64 years and adults aged 65 years or older: an integration of physical activity, sedentary behaviour, and sleep. Appl Physiol Nut Metab. 2020;45(10 (suppl. 2)):S57–S102. doi:10.1139/apnm-2020-0467 10.1139/apnm-2020-0467 33054332

[11] Bull FC , Al-Ansari SS, Biddle S, et al. World Health Organization 2020 guidelines on physical activity and sedentary behaviour. Br J Sports Med. 2020;54(24):1451–1462. doi:10.1136/bjsports-2020-102955 33239350 10.1136/bjsports-2020-102955 PMC7719906

[12] Committee PAGA. 2018 Physical Activity Guidelines Advisory Committee Scientific Report. US Department of Health and Human Services; 2018.

[13] López-Valenciano A , Mayo X, Liguori G, Copeland RJ, Lamb M, Jimenez A. Changes in sedentary behaviour in European Union adults between 2002 and 2017. BMC Public Health. 2020;20(1):1206. doi:10.1186/s12889-020-09293-1 32843022 10.1186/s12889-020-09293-1 PMC7448983

[14] Woessner MN , Tacey A, Levinger-Limor A, Parker AG, Levinger P, Levinger I. The evolution of technology and physical inactivity: the good, the bad, and the way forward. Front Public Health. 2021;9:655491. doi:10.3389/fpubh.2021.655491 34123989 10.3389/fpubh.2021.655491 PMC8193221

[15] Healy GN , Matthews CE, Dunstan DW, Winkler EA, Owen N. Sedentary time and cardio-metabolic biomarkers in US adults: NHANES 2003–06. Eur Heart J. 2011;32(5):590–597. doi:10.1093/eurheartj/ehq451 21224291 10.1093/eurheartj/ehq451 PMC3634159

[16] Harvey JA , Chastin SF, Skelton DA. How sedentary are older people? A systematic review of the amount of sedentary behavior. J Aging Phys Act. 2015;23(3):471–487. doi:10.1123/japa.2014-0164 25387160 10.1123/japa.2014-0164

[17] Jefferis BJ , Parsons TJ, Sartini C, et al. Does total volume of physical activity matter more than pattern for onset of CVD? A prospective cohort study of older British men. Int J Cardiol. 2019;278:267–272. doi:10.1016/j.ijcard.2018.12.024 30578094 10.1016/j.ijcard.2018.12.024 PMC6350006

[18] Bellettiere J , LaMonte MJ, Evenson KR, et al. Sedentary behavior and cardiovascular disease in older women: the Objective Physical Activity and Cardiovascular Health (OPACH) Study. Circulation. 2019;139(8):1036–1046. doi:10.1161/CIRCULATIONAHA.118.035312 31031411 10.1161/CIRCULATIONAHA.118.035312 PMC6481298

[19] Diaz KM , Howard VJ, Hutto B, et al. Patterns of sedentary behavior and mortality in U.S. middle-aged and older adults: a national cohort study. Ann Intern Med. 2017;167(7):465–475. doi:10.7326/M17-0212 28892811 10.7326/M17-0212 PMC5961729

[20] Jefferis BJ , Parsons TJ, Sartini C, et al. Objectively measured physical activity, sedentary behaviour and all-cause mortality in older men: does volume of activity matter more than pattern of accumulation? Br J Sports Med. 2019;53(16):1013–1020. doi:10.1136/bjsports-2017-098733 29440040 10.1136/bjsports-2017-098733 PMC6691867

[21] Boerema ST , van Velsen L, Vollenbroek MM, Hermens HJ. Pattern measures of sedentary behaviour in adults: a literature review. Digit Health. 2020;6:2055207620905418. doi:10.1177/2055207620905418 32095261 10.1177/2055207620905418 PMC7013117

[22] Chastin SF , Winkler EA, Eakin EG, et al. Sensitivity to change of objectively-derived measures of sedentary behavior. Meas Phys Educ Exerc Sci. 2015;19(3):138–147. doi:10.1080/1091367X.2015.1050592

[23] Marmot M , Brunner E. Cohort profile: the Whitehall II study. Int J Epidemiol. 2005;34(2):251–256. doi:10.1093/ije/dyh372 15576467 10.1093/ije/dyh372

[24] Migueles JH , Rowlands AV, Huber F, Sabia S, van Hees VT. GGIR: a research community-driven open source R package for generating physical activity and sleep outcomes from multi-day raw accelerometer data. J Meas Phys Behav. 2019;2(3):188–196. doi:10.1123/jmpb.2018-0063

[25] van Hees VT , Gorzelniak L, Dean León EC, et al. Separating movement and gravity components in an acceleration signal and implications for the assessment of human daily physical activity. PLoS One. 2013;8(4):e61691. doi:10.1371/journal.pone.0061691 23626718 10.1371/journal.pone.0061691 PMC3634007

[26] van Hees VT , Sabia S, Anderson KN, et al. A novel, open access method to assess sleep duration using a wrist-worn accelerometer. PLoS One. 2015;10(11):e0142533. doi:10.1371/journal.pone.0142533 26569414 10.1371/journal.pone.0142533 PMC4646630

[27] Menai M , van Hees VT, Elbaz A, Kivimaki M, Singh-Manoux A, Sabia S. Accelerometer assessed moderate-to-vigorous physical activity and successful ageing: results from the Whitehall II study. Sci Rep. 2017;7(1):45772. doi:10.1038/srep45772 10.1038/srep45772 PMC5377945 28367987

[28] Hildebrand M , Hansen BH, van Hees VT, Ekelund U. Evaluation of raw acceleration sedentary thresholds in children and adults. Scand J Med Sci Sports. 2017;27(12):1814–1823. doi:10.1111/sms.12795 27878845 10.1111/sms.12795

[29] Rowlands AV , Mirkes EM, Yates T, et al. Accelerometer-assessed physical activity in epidemiology: are monitors equivalent? Med Sci Sports Exerc. 2018;50(2):257–265. doi:10.1249/MSS.0000000000001435 28976493 10.1249/MSS.0000000000001435

[30] Fraysse F , Post D, Eston R, Kasai D, Rowlands AV, Parfitt G. Physical activity intensity cut-points for wrist-worn GENEActiv in older adults. Front Sports Act Living. 2020;2:579278. doi:10.3389/fspor.2020.5792788 33521631 10.3389/fspor.2020.579278 PMC7843957

[31] Migueles JH , Cadenas-Sanchez C, Alcantara JMA, et al. Calibration and cross-validation of accelerometer cut-points to classify sedentary time and physical activity from hip and non-dominant and dominant wrists in older adults. Sensors (Basel, Switzerland). 2021;21(10). doi:10.3390/s21103326 10.3390/s21103326 PMC8150960 34064790

[32] Chastin SF , Granat MH. Methods for objective measure, quantification and analysis of sedentary behaviour and inactivity. Gait Posture. 2010;31(1):82–86. doi:10.1016/j.gaitpost.2009.09.002 19854651 10.1016/j.gaitpost.2009.09.002

[33] J D , Leroux A, Urbanek J, et al. Patterns of sedentary and active time accumulation are associated with mortality in US adults: the NHANES study. biorXiv. 2017:182337. doi:10.1101/182337

[34] Chastin SFM , Ferriolli E, Stephens NA, Fearon KC, Greig C. Relationship between sedentary behaviour, physical activity, muscle quality and body composition in healthy older adults. Age Ageing. 2012;41(1):111–114. doi:10.1093/ageing/afr075 21749993 10.1093/ageing/afr075

[35] Lyden K , Kozey Keadle SL, Staudenmayer JW, Freedson PS. Validity of two wearable monitors to estimate breaks from sedentary time. Med Sci Sports Exerc. 2012;44(11):2243–2252. doi:10.1249/MSS.0b013e318260c477 22648343 10.1249/MSS.0b013e318260c477 PMC3475768

[36] Kivimäki M , Batty GD, Singh-Manoux A, Britton A, Brunner EJ, Shipley MJ. Validity of cardiovascular disease event ascertainment using linkage to UK hospital records. Epidemiology (Cambridge, Mass). 2017;28(5):735–739. doi:10.1097/EDE.0000000000000688 10.1097/EDE.0000000000000688 PMC5540351 28570383

[37] Harrell FE Jr . Regression Modeling Strategies: With Applications to Linear Models, Logistic and Ordinal Regression, and Survival Analysis. Springer; 2015.

[38] Pandey A , Salahuddin U, Garg S, et al. Continuous dose–response association between sedentary time and risk for cardiovascular disease: a meta-analysis. JAMA Cardiol. 2016;1(5):575–583. doi:10.1001/jamacardio.2016.1567 27434872 10.1001/jamacardio.2016.1567

[39] Bailey DP , Hewson DJ, Champion RB, Sayegh SM. Sitting time and risk of cardiovascular disease and diabetes: a systematic review and meta-analysis. Am J Prev Med. 2019;57(3):408–416. doi:10.1016/j.amepre.2019.04.015 31377090 10.1016/j.amepre.2019.04.015

[40] Dempsey PC , Strain T, Khaw KT, Wareham NJ, Brage S, Wijndaele K. Prospective associations of accelerometer-measured physical activity and sedentary time with incident cardiovascular disease, cancer, and all-cause mortality. Circulation. 2020;141(13):1113–1115. doi:10.1161/CIRCULATIONAHA.119.043030 32223676 10.1161/CIRCULATIONAHA.119.043030

[41] Dohrn IM , Welmer AK, Hagströmer M. Accelerometry-assessed physical activity and sedentary time and associations with chronic disease and hospital visits—a prospective cohort study with 15 years follow-up. Int J Behav Nutr Phys Act. 2019;16(1):125. doi:10.1186/s12966-019-0878-2 31818303 10.1186/s12966-019-0878-2 PMC6902520

[42] Yerramalla MS , McGregor DE, van Hees VT, et al. Association of daily composition of physical activity and sedentary behaviour with incidence of cardiovascular disease in older adults. Int J Behav Nutr Phys Act. 2021;18(1):83. doi:10.1186/s12966-021-01157-0 34247647 10.1186/s12966-021-01157-0 PMC8273960

[43] Carson V , Wong SL, Winkler E, Healy GN, Colley RC, Tremblay MS. Patterns of sedentary time and cardiometabolic risk among Canadian adults. Prev Med. 2014;65:23–27. doi:10.1016/j.ypmed.2014.04.005 24732719 10.1016/j.ypmed.2014.04.005

[44] Bellettiere J , Winkler EAH, Chastin SFM, et al. Associations of sitting accumulation patterns with cardio-metabolic risk biomarkers in Australian adults. PLoS One. 2017;12(6):e0180119. doi:10.1371/journal.pone.0180119 28662164 10.1371/journal.pone.0180119 PMC5491133

[45] Diaz KM , Duran AT, Colabianchi N, Judd SE, Howard VJ, Hooker SP. Potential effects on mortality of replacing sedentary time with short sedentary bouts or physical activity: a national cohort study. Am J Epidemiol. 2019;188(3):537–544. doi:10.1093/aje/kwy271 30551177 10.1093/aje/kwy271 PMC6395167

[46] Kalache A , Kickbusch I. A global strategy for healthy ageing. World Health. 1997;50(4):4–5. https://apps.who.int/iris/handle/10665/330616

[47] Batty GD , Shipley M, Tabák A, et al. Generalizability of occupational cohort study findings. Epidemiology (Cambridge, Mass). 2014;25(6):932–933. doi:10.1097/EDE.0000000000000184 10.1097/EDE.0000000000000184 25265141

