# Supplementary material for: Objectively Measured Total Sedentary Time and Pattern of Sedentary Accumulation in Older Adults: Associations With Incident Cardiovascular Disease and All-Cause Mortality
Source: J Gerontol A Biol Sci Med Sci. 2022 Jan 30;77(4):842–50. doi: 10.1093/gerona/glac023 (PMC8974336; doi:10.1093/gerona/glac023)
Supplement: glac023_suppl_Supplementary_Material [file glac023_suppl_supplementary_material.docx]

**SUPPLEMENT**

**TABLE OF CONTENTS**

[**eMETHODS** 2](#_Toc90048510)

[Metrics of sedentary accumulation pattern 2](#_Toc90048511)

[**SUPPLEMENTARY TABLES** 3](#_Toc90048512)

[**eTable 1** Characteristics of participants included in the study sample and those excluded 3](#_Toc90048513)

[**eTable 2** Correlation matrix of total sedentary time, sedentary accumulation patterns, and MVPA duration 4](#_Toc90048514)

[**eTable 3** Associations of total sedentary time and sedentary accumulation patterns with incident CVD adjusted for sociodemographic, behavioural factors and MVPA 5](#_Toc90048515)

[**eTable 4** Association of MVPA with incident CVD and all-cause mortality 6](#_Toc90048516)

[**eTable 5** Associations of total sedentary time and sedentary accumulation patterns with all-cause mortality among participants aged <74 years (N total = 3001, N events = 114, mean follow-up (SD) = 6.5 (0.7) years, mean age (SD) = 66.7 (3.6) years) 7](#_Toc90048517)

[**eTable 6** Associations of total sedentary time and sedentary accumulation patterns with all-cause mortality among participants aged ≥74 years (N total = 990, N events = 146, mean follow-up (SD) = 6.2 (1.2) years, mean age (SD) = 77.5 (2.2) years) 8](#_Toc90048518)

[**eTable 7** Differences in the means of total sedentary time and sedentary accumulation patterns by age group 9](#_Toc90048519)

[**eTable 8** Associations of total sedentary time and sedentary accumulation patterns with incident CVD using a 2-year wash-out period (N total = 3233, N events = 211, mean follow-up (SD) = 6.3 (0.9) years) 10](#_Toc90048520)

[**eTable 9** Associations of total sedentary time and sedentary accumulation patterns with all-cause mortality using a 2-year wash-out period (N total = 3946, N events = 215, mean follow-up (SD) = 6.4 (0.6) years) 11](#_Toc90048521)

[**eTable 10** Associations of total sedentary time and sedentary accumulation patterns with all-cause mortality using a 2-year wash-out period stratified by age 12](#_Toc90048522)

[**eTable 11** Associations of total sedentary time and sedentary accumulation patterns with incident CVD and all-cause mortality adjusting for MVPA as a continuous variable in the fully adjusted model 13](#_Toc90048523)

[**eTable 12** Associations of total sedentary time and sedentary accumulation patterns with all-cause mortality stratified by age adjusting for MVPA as a continuous variable in the fully adjusted model 14](#_Toc90048524)

[**SUPPLEMENTARY FIGURES** 15](#_Toc90048525)

[**eFigure 1** Participant flow chart 15](#_Toc90048526)

[**eFigure 2** Associations of total sedentary time and sedentary accumulation patterns with all-cause mortality stratified by median age of the sample population 16](#_Toc90048527)

# **eMETHODS**

## Metrics of sedentary accumulation pattern

1. Mean sedentary bout duration, the total sedentary time divided by number of sedentary bouts, with higher value indicating less fragmentation of sedentary time.^1^
2. Time in prolonged sedentary bouts, the time spent in SB bouts lasting at least 30 minutes, with higher value denoting more and/or longer long periods of uninterrupted sedentary time.
3. Gini index, a measure of the variability of the distribution of sedentary bout lengths, ranging from 0 to 1.^2,3^ A Gini index of 0 denotes that sedentary bouts of all lengths contributed equally to total sedentary time, while 1 indicates that total sedentary time accumulates through few numbers of sedentary bouts of longer length.
4. Number of sedentary breaks, with a break defined as an interruption of a SB bout. Higher number of sedentary breaks corresponds to a more fragmented pattern of SB.^1^
5. Breaks per sedentary hour, sometimes referred to as either fragmentation index ^4^ or break-rate,^5^ is the ratio of the number of sedentary breaks divided by the total sedentary time in hours.
6. Alpha, sometimes referred to as Power law scaling exponent characterizes the distribution of sedentary bout lengths.^2^ A lower value of Alpha indicates that sedentary time accumulates mainly in smaller proportion of longer sedentary bouts, while a higher value corresponds to accumulation of sedentary time in a larger proportion of short duration bouts.
7. Transition Probability from Sedentary to LIPA or MVPA state, defined as proportion of total sedentary time before LIPA (or MVPA) to total sedentary time divided by mean sedentary bout duration. These metrics were calculated based on the work of Junrui Di and colleague.^3^ Larger values of the 2 measures represent more switching or transitioning from sedentary to LIPA and MVPA states, respectively.^3^

**References**

1.Healy GN, Dunstan DW, Salmon J, et al. Breaks in sedentary time: beneficial associations with metabolic risk. *Diabetes care*. Apr 2008;31(4):661-6. doi:10.2337/dc07-2046

2.Chastin SF, Granat MH. Methods for objective measure, quantification and analysis of sedentary behaviour and inactivity. *Gait & posture*. Jan 2010;31(1):82-6. doi:10.1016/j.gaitpost.2009.09.002

3.Di J, Leroux A, Urbanek J, et al. Patterns of sedentary and active time accumulation are associated with mortality in US adults: The NHANES study. *bioRxiv*. 2017:182337. doi:10.1101/182337

4.Chastin SFM, Ferriolli E, Stephens NA, Fearon KC, Greig C. Relationship between sedentary behaviour, physical activity, muscle quality and body composition in healthy older adults. *Age and ageing*. 2012;41(1):111-114.

5.Lyden K, Kozey Keadle SL, Staudenmayer JW, Freedson PS. Validity of two wearable monitors to estimate breaks from sedentary time. *Medicine and science in sports and exercise*. Nov 2012;44(11):2243-52. doi:10.1249/MSS.0b013e318260c477

**SUPPLEMENTARY TABLES**

**eTable 1** Characteristics of participants included in the study sample and those excluded

|  | **Included in the study sample** | |  |
| --- | --- | --- | --- |
| **Characteristics** | **No** | **Yes** | **P value** |
| N (row %) | 889 (18.2) | 3991 (81.8) |  |
| Age (years), M (SD) | 68.9 (5.6) | 69.4 (5.7) | 0.03 |
| Women | 298 (33.5) | 1030 (25.8) | <0.001 |
| Non-white | 93 (10.5) | 395 (7.4) | <0.01 |
| Married/cohabitating | 654 (73.6) | 2981 ( 74.7) | 0.49 |
| University or higher degree | 325 (36.6) | 1238 (31.0) | <0.01 |
| Low occupational position | 443 (49.8) | 2031 (50.9) | 0.57 |

Abbreviations: M, mean; SD, standard deviation.

Values are N (col %) unless otherwise stated.

**eTable 2** Correlation matrix of total sedentary time, sedentary accumulation patterns, and MVPA duration

|  | (1) | (2) | (3) | (4) | (5) | (6) | (7) | (9) | (10) | (11) |
| --- | --- | --- | --- | --- | --- | --- | --- | --- | --- | --- |
| 1. Total sedentary time | 1.00 |  |  |  |  |  |  |  |  |  |
| 1. Mean sedentary bout duration | 0.62 | 1.00 |  |  |  |  |  |  |  |  |
| 1. Time in prolonged (≥30 min) sedentary bouts | 0.88 | 0.78 | 1.00 |  |  |  |  |  |  |  |
| 1. Gini index | 0.45 | 0.41 | 0.70 | 1.00 |  |  |  |  |  |  |
| 1. Number of sedentary breaks | -0.51 | -0.76 | -0.78 | -0.52 | 1.00 |  |  |  |  |  |
| 1. Breaks per sedentary hour | -0.80 | -0.74 | -0.91 | -0.58 | 0.90 | 1.00 |  |  |  |  |
| 1. Alpha | -0.77 | -0.71 | -0.77 | -0.24 | 0.82 | 0.92 | 1.00 |  |  |  |
| 1. Transition probability from sedentary to LIPA state | -0.77 | -0.73 | -0.88 | -0.56 | 0.90 | 0.99 | 0.92 | 1.00 |  |  |
| 1. Transition probability from sedentary to MVPA state | -0.52 | -0.36 | -0.53 | -0.43 | 0.35 | 0.49 | 0.40 | 0.37 | 1.00 |  |
| (10) MVPA duration | -0.65 | -0.39 | -0.58 | -0.37 | 0.27 | 0.49 | 0.43 | 0.42 | 0.67 | 1.00 |

Abbreviations: PA, physical activity; LIPA, light intensity physical activity; MVPA, moderate-to-vigorous physical activity.

**eTable 3** Associations of total sedentary time and sedentary accumulation patterns with incident CVD adjusted for sociodemographic, behavioural factors and MVPA

|  |  | **HR (95% CI)** | |
| --- | --- | --- | --- |
|  |  | **Adjusted for behavioural and sociodemographic factors^a^** | **Additionally adjusted for MVPA^b^** |
| Total sedentary time |  | 1.20 (1.05-1.37) | 1.08 (0.93-1.26) |
| **Sedentary accumulation patterns^c^** |  |  |  |
| (1) Mean sedentary bout duration |  | 1.19 (1.08-1.30) | 1.12 (1.00-1.24) |
| (2) Time in prolonged (≥30 min) sedentary bouts |  | 1.19 (1.06-1.33) | 1.10 (0.96-1.25) |
| (3) Gini index |  | 1.07 (0.95-1.21) | 1.02 (0.90-1.15) |
| (4) Number of sedentary breaks |  | 0.86 (0.77-0.97) | 0.92 (0.81-1.03) |
| (5) Breaks per sedentary hour |  | 0.86 (0.76-0.97) | 0.93 (0.81-1.05) |
| (6) Alpha |  | 0.85 (0.75-0.95) | 0.90 (0.80-1.03) |
| Transition probability from |  |  |  |
| (7) sedentary to LIPA state |  | 0.87 (0.78-0.99) | 0.94 (0.83-1.06) |
| (8) sedentary to MVPA state |  | 0.82 (0.70-0.95) | 0.88 (0.76-1.03) |

Abbreviations: CVD, cardiovascular disease; CI, confidence interval; HR, hazard ratio; PA, physical activity; LIPA, light intensity physical activity;

MVPA, moderate-to-vigorous physical activity; SD, standard deviation.

^a^Models adjusted for age (time-scale), sex, ethnicity, education, occupation position, marital status, total waking day duration, smoking status, alcohol consumption and fruits and vegetables consumption.

^b^Models additionally adjusted for MVPA recommendation.

^c^Metrics are standardized based on sample mean & SD resulting in HRs corresponding to one SD higher value. For metrics 1-3, an increase of 1-SD corresponds to less favourable sedentary accumulation pattern. For metrics 4-8, an increase of 1-SD corresponds to more favourable sedentary accumulation pattern. 1 SD represents 100.2 minutes for total sedentary time, 6.1 minutes for mean sedentary bout duration, 143.2 minutes for time in prolonged (≥30 min) sedentary bouts, 0.036 for Gini Index, 16.0 for number of sedentary breaks, 6.2 for breaks per sedentary hour, 0.127 for Alpha, and 3.1%, and 0.5% for transition probability from sedentary to LIPA and MVPA states, respectively.

**eTable 4** Association of MVPA with incident CVD and all-cause mortality

|  | **HR (95% CI)** | |
| --- | --- | --- |
|  | **Incident CVD** | **All-cause mortality** |
| Following recommendation of 150 min per week of MVPA | |  |
| No | 1.00 | 1.00 |
| Yes | 0.69 (0.52-0.92) | 0.59 (0.44-0.78) |
| Daily time in MVPA, per 1 SD (38.6 min) | 0.90 (0.78-1.04) | 0.70 (0.59-0.85) |

Abbreviations: CI, confidence interval; HR, hazard ratio; MVPA, moderate-to-vigorous physical activity; SD, standard deviation.

All models adjusted for age (time-scale), sex, ethnicity, education, occupation position, marital status, total waking day duration, smoking status, alcohol consumption, fruits and vegetables consumption, prevalent diabetes, BMI, hypertension, hyperlipidaemia, and morbidity index.

**eTable 5** Associations of total sedentary time and sedentary accumulation patterns with all-cause mortality among participants aged <74 years (N total = 3001, N events = 114, mean follow-up (SD) = 6.5 (0.7) years, mean age (SD) = 66.7 (3.6) years)

|  | **HR (95% CI)** | | | |
| --- | --- | --- | --- | --- |
|  | **Adjusted for sociodemographic factors^a^** | **Additionally adjusted for behavioural factors^b^** | **Additionally adjusted for health-related factors^c^** | **Additionally adjusted for MVPA^d^** |
| Total sedentary time | 1.56 (1.26-1.94) | 1.55 (1.24-1.92) | 1.56 (1.25-1.95) | 1.45 (1.12-1.88) |
| **Sedentary accumulation patterns^e^** |  |  |  |  |
| (1) Mean sedentary bout duration | 1.11 (1.02-1.20) | 1.11 (1.01-1.21) | 1.11 (1.02-1.22) | 1.06 (0.96-1.18) |
| (2) Time in prolonged (≥30 min) sedentary bouts | 1.49 (1.25-1.77) | 1.47 (1.23-1.76) | 1.48 (1.23-1.77) | 1.39 (1.13-1.71) |
| (3) Gini index | 1.38 (1.13-1.67) | 1.37 (1.13-1.66) | 1.36 (1.12-1.66) | 1.30 (1.06-1.58) |
| (4) Number of sedentary breaks | 0.71 (0.59-0.85) | 0.71 (0.59-0.86) | 0.72 (0.60-0.87) | 0.77 (0.63-0.94) |
| (5) Breaks per sedentary hour | 0.67 (0.55-0.82) | 0.68 (0.55-0.83) | 0.68 (0.55-0.83) | 0.73 (0.59-0.91) |
| (6) Alpha | 0.73 (0.60-0.88) | 0.73 (0.60-0.89) | 0.74 (0.61-0.90) | 0.80 (0.65-0.98) |
| Transition probability from |  |  |  |  |
| (7) sedentary to LIPA state | 0.69 (0.56-0.85) | 0.70 (0.57-0.86) | 0.70 (0.57-0.86) | 0.75 (0.61-0.94) |
| (8) sedentary to MVPA state | 0.69 (0.53-0.89) | 0.69 (0.54-0.89) | 0.70 (0.54-0.90) | 0.77 (0.59-1.00) |

Abbreviations: CI, confidence interval; HR, hazard ratio; PA, physical activity; LIPA, light intensity physical activity;

MVPA, moderate-to-vigorous physical activity; SD, standard deviation.

^a^Models adjusted for age (time-scale), sex, ethnicity, education, occupation position, marital status and total waking day duration.

^b^Models additionally adjusted for smoking status, alcohol consumption and fruits and vegetables consumption.

^c^Models additionally adjusted for prevalent diabetes, BMI, hypertension, hyperlipidaemia and morbidity index.

^d^Models additionally adjusted for MVPA recommendation.

^e^Metrics are standardized based on sample mean & SD resulting in HRs corresponding to one SD higher value. For metrics 1-3, an increase of 1-SD corresponds to less favourable sedentary accumulation pattern. For metrics 4-8, an increase of 1-SD corresponds to more favourable sedentary accumulation pattern. 1 SD represents 100.2 minutes for total sedentary time, 6.1 minutes for mean sedentary bout duration, 143.2 minutes for time in prolonged (≥30 min) sedentary bouts, 0.036 for Gini Index, 16.0 for number of sedentary breaks, 6.2 for breaks per sedentary hour, 0.127 for Alpha, and 3.1%, and 0.5% for transition probability from sedentary to LIPA and MVPA states, respectively.

**eTable 6** Associations of total sedentary time and sedentary accumulation patterns with all-cause mortality among participants aged ≥74 years (N total = 990, N events = 146, mean follow-up (SD) = 6.2 (1.2) years, mean age (SD) = 77.5 (2.2) years)

|  | **HR (95% CI)** | | | |
| --- | --- | --- | --- | --- |
|  | **Adjusted for sociodemographic factors^a^** | **Additionally adjusted for behavioural factors^b^** | **Additionally adjusted for health-related factors^c^** | **Additionally adjusted for MVPA^d^** |
| Total sedentary time | 1.22 (1.01-1.47) | 1.19 (0.98-1.43) | 1.12 (0.92-1.36) | 0.95 (0.76-1.20) |
| **Sedentary accumulation patterns^e^** |  |  |  |  |
| (1) Mean sedentary bout duration | 1.08 (0.99-1.19) | 1.06 (0.97-1.17) | 1.04 (0.94-1.15) | 1.00 (0.89-1.12) |
| (2) Time in prolonged (≥30 min) sedentary bouts | 1.14 (0.98-1.33) | 1.12 (0.96-1.31) | 1.07 (0.91-1.26) | 0.96 (0.80-1.15) |
| (3) Gini index | 0.98 (0.83-1.17) | 0.98 (0.83-1.17) | 0.96 (0.80-1.14) | 0.92 (0.77-1.09) |
| (4) Number of sedentary breaks | 0.93 (0.80-1.08) | 0.94 (0.81-1.10) | 0.98 (0.83-1.15) | 1.04 (0.89-1.23) |
| (5) Breaks per sedentary hour | 0.90 (0.76-1.07) | 0.92 (0.78-1.09) | 0.97 (0.81-1.16) | 1.08 (0.89-1.30) |
| (6) Alpha | 0.88 (0.75-1.03) | 0.89 (0.76-1.05) | 0.93 (0.79-1.10) | 1.02 (0.85-1.23) |
| Transition probability from |  |  |  |  |
| (7) sedentary to LIPA state | 0.92 (0.78-1.08) | 0.94 (0.79-1.10) | 0.98 (0.82-1.16) | 1.08 (0.90-1.29) |
| (8) sedentary to MVPA state | 0.69 (0.51-0.93) | 0.71 (0.53-0.97) | 0.79 (0.58-1.08) | 0.93 (0.67-1.29) |

Abbreviations: CI, confidence interval; HR, hazard ratio; PA, physical activity; LIPA, light intensity physical activity;

MVPA, moderate-to-vigorous physical activity; SD, standard deviation.

^a^Models adjusted for age (time-scale), sex, ethnicity, education, occupation position, marital status and total waking day duration.

^b^Models additionally adjusted for smoking status, alcohol consumption and fruits and vegetables consumption.

^c^Models additionally adjusted for prevalent diabetes, BMI, hypertension, hyperlipidaemia and morbidity index.

^d^Models additionally adjusted for MVPA recommendation.

^e^Metrics are standardized based on sample mean & SD resulting in HRs corresponding to one SD higher value. For metrics 1-3, an increase of 1-SD corresponds to less favourable sedentary accumulation pattern. For metrics 4-8, an increase of 1-SD corresponds to more favourable sedentary accumulation pattern. 1 SD represents 100.2 minutes for total sedentary time, 6.1 minutes for mean sedentary bout duration, 143.2 minutes for time in prolonged (≥30 min) sedentary bouts, 0.036 for Gini Index, 16.0 for number of sedentary breaks, 6.2 for breaks per sedentary hour, 0.127 for Alpha, and 3.1%, and 0.5% for transition probability from sedentary to LIPA and MVPA states, respectively.

**eTable 7** Differences in the means of total sedentary time and sedentary accumulation patterns by age group

|  |  | **Age<74 years** | **Age≥74 years** | **P value^a^** |
| --- | --- | --- | --- | --- |
| N |  | 3001 | 990 |  |
| Total sedentary time |  | 707.0 (97.8) | 750.7 (100.3) | <0.001 |
| **Sedentary accumulation patterns^b^** |  |  |  |  |
| (1) Mean sedentary bout duration |  | 10.9 (5.2) | 13.1 (7.9) | <0.001 |
| (2) Time in prolonged (≥30 min) sedentary bouts |  | 366.8 (135.8) | 439.2 (151.2) | <0.001 |
| (3) Gini index |  | 0.67 (0.04) | 0.69 (0.04) | <0.001 |
| (4) Number of sedentary breaks |  | 73.0 (15.2) | 68.4 (17.7) | <0.001 |
| (5) Breaks per sedentary hour |  | 6.4 (1.9) | 5.7 (2.0) | <0.001 |
| (6) Alpha |  | 1.76 (0.17) | 1.73 (0.10) | <0.001 |
| Transition probability (%) from |  |  |  |  |
| (7) sedentary to LIPA state |  | 10.0 (3.0) | 9.1 (3.1) | <0.001 |
| (8) sedentary to MVPA state |  | 0.61 (0.50) | 0.32 (0.33) | <0.001 |

^a^T-test to compare the differences in mean by age.

^b^For metrics 1-3, an increase of value corresponds to less favourable sedentary accumulation pattern. For metrics 4-8, an increase of value corresponds to more favourable sedentary accumulation pattern.

**eTable 8** Associations of total sedentary time and sedentary accumulation patterns with incident CVD using a 2-year wash-out period (N total = 3233, N events = 211, mean follow-up (SD) = 6.3 (0.9) years)

|  | **HR (95% CI)** | | | |
| --- | --- | --- | --- | --- |
|  | **Adjusted for sociodemographic factors^a^** | **Additionally adjusted for behavioural factors^b^** | **Additionally adjusted for health-related factors^c^** | **Additionally adjusted for MVPA^d^** |
| Total sedentary time | 1.12 (0.96-1.30) | 1.10 (0.95-1.28) | 1.01 (0.86-1.18) | 0.94 (0.79-1.13) |
| **Sedentary accumulation patterns^e^** |  |  |  |  |
| (1) Mean sedentary bout duration | 1.13 (1.01-1.27) | 1.14 (1.01-1.28) | 1.08 (0.95-1.23) | 1.05 (0.91-1.21) |
| (2) Time in prolonged (≥30 min) sedentary bouts | 1.09 (0.95-1.25) | 1.09 (0.95-1.25) | 1.01 (0.87-1.17) | 0.96 (0.81-1.13) |
| (3) Gini index | 0.94 (0.82-1.07) | 0.95 (0.83-1.10) | 0.91 (0.79-1.05) | 0.89 (0.77-1.02) |
| (4) Number of sedentary breaks | 0.94 (0.82-1.08) | 0.94 (0.82-1.08) | 1.00 (0.86-1.15) | 1.03 (0.89-1.19) |
| (5) Breaks per sedentary hour | 0.94 (0.82-1.09) | 0.95 (0.83-1.09) | 1.02 (0.89-1.18) | 1.07 (0.92-1.25) |
| (6) Alpha | 0.89 (0.77-1.02) | 0.90 (0.78-1.03) | 0.96 (0.83-1.11) | 0.99 (0.85-1.15) |
| Transition probability from |  |  |  |  |
| (7) sedentary to LIPA state | 0.95 (0.83-1.09) | 0.96 (0.83-1.10) | 1.03 (0.89-1.18) | 1.07 (0.92-1.24) |
| (8) sedentary to MVPA state | 0.93 (0.79-1.10) | 0.94 (0.80-1.11) | 1.00 (0.85-1.18) | 1.05 (0.88-1.24) |

Abbreviations: CVD, cardiovascular disease; CI, confidence interval; HR, hazard ratio; PA, physical activity; LIPA, light intensity physical activity;

MVPA, moderate-to-vigorous physical activity; SD, standard deviation.

^a^Models adjusted for age (time-scale), sex, ethnicity, education, occupation position, marital status and total waking day duration.

^b^Models additionally adjusted for smoking status, alcohol consumption and fruits and vegetables consumption.

^c^Models additionally adjusted for prevalent diabetes, BMI, hypertension, hyperlipidaemia and morbidity index.

^d^Models additionally adjusted for MVPA recommendation.

^e^Metrics are standardized based on sample mean & SD resulting in HRs corresponding to one SD higher value. For metrics 1-3, an increase of 1-SD corresponds to less favourable sedentary accumulation pattern. For metrics 4-8, an increase of 1-SD corresponds to more favourable sedentary accumulation pattern. 1 SD represents 100.2 minutes for total sedentary time, 6.1 minutes for mean sedentary bout duration, 143.2 minutes for time in prolonged (≥30 min) sedentary bouts, 0.036 for Gini Index, 16.0 for number of sedentary breaks, 6.2 for breaks per sedentary hour, 0.127 for Alpha, and 3.1%, and 0.5% for transition probability from sedentary to LIPA and MVPA states, respectively.

**eTable 9** Associations of total sedentary time and sedentary accumulation patterns with all-cause mortality using a 2-year wash-out period (N total = 3946, N events = 215, mean follow-up (SD) = 6.4 (0.6) years)

|  | **HR (95% CI)** | | | |
| --- | --- | --- | --- | --- |
|  | **Adjusted for sociodemographic factors^a^** | **Additionally adjusted for behavioural factors^b^** | **Additionally adjusted for health-related factors^c^** | **Additionally adjusted for MVPA^d^** |
| Total sedentary time | 1.39 (1.19-1.62) | 1.37 (1.17-1.60) | 1.33 (1.13-1.56) | 1.21 (1.00-1.46) |
| **Sedentary accumulation patterns^e^** |  |  |  |  |
| (1) Mean sedentary bout duration | 1.11 (1.04-1.18) | 1.10 (1.02-1.17) | 1.09 (1.01-1.17) | 1.04 (0.96-1.13) |
| (2) Time in prolonged (≥30 min) sedentary bouts | 1.28 (1.13-1.45) | 1.27 (1.11-1.44) | 1.23 (1.08-1.41) | 1.13 (0.97-1.32) |
| (3) Gini index | 1.12 (0.97-1.29) | 1.11 (0.97-1.28) | 1.08 (0.94-1.25) | 1.03 (0.89-1.19) |
| (4) Number of sedentary breaks | 0.83 (0.73-0.94) | 0.84 (0.74-0.95) | 0.86 (0.75-0.98) | 0.92 (0.80-1.05) |
| (5) Breaks per sedentary hour | 0.79 (0.69-0.91) | 0.80 (0.69-0.92) | 0.82 (0.71-0.95) | 0.89 (0.76-1.05) |
| (6) Alpha | 0.79 (0.69-0.90) | 0.80 (0.70-0.91) | 0.82 (0.71-0.94) | 0.88 (0.76-1.03) |
| Transition probability from |  |  |  |  |
| (7) sedentary to LIPA state | 0.81 (0.70-0.93) | 0.82 (0.71-0.94) | 0.84 (0.73-0.97) | 0.91 (0.78-1.06) |
| (8) sedentary to MVPA state | 0.70 (0.57-0.87) | 0.71 (0.58-0.88) | 0.75 (0.61-0.93) | 0.84 (0.67-1.05) |

Abbreviations: CI, confidence interval; HR, hazard ratio; PA, physical activity; LIPA, light intensity physical activity;

MVPA, moderate-to-vigorous physical activity; SD, standard deviation.

^a^Models adjusted for age (time-scale), sex, ethnicity, education, occupation position, marital status and total waking day duration.

^b^Models additionally adjusted for smoking status, alcohol consumption and fruits and vegetables consumption.

^c^Models additionally adjusted for prevalent diabetes, BMI, hypertension, hyperlipidaemia and morbidity index.

^d^Models additionally adjusted for MVPA recommendation.

^e^Metrics are standardized based on sample mean & SD resulting in HRs corresponding to one SD higher value. For metrics 1-3, an increase of 1-SD corresponds to less favourable sedentary accumulation pattern. For metrics 4-8, an increase of 1-SD corresponds to more favourable sedentary accumulation pattern. 1 SD represents 100.2 minutes for total sedentary time, 6.1 minutes for mean sedentary bout duration, 143.2 minutes for time in prolonged (≥30 min) sedentary bouts, 0.036 for Gini Index, 16.0 for number of sedentary breaks, 6.2 for breaks per sedentary hour, 0.127 for Alpha, and 3.1%, and 0.5% for transition probability from sedentary to LIPA and MVPA states, respectively.

**eTable 10** Associations of total sedentary time and sedentary accumulation patterns with all-cause mortality using a 2-year wash-out period stratified by age

|  | **HR (95% CI)** | | | | |
| --- | --- | --- | --- | --- | --- |
|  | **Age<74 years^a^** | |  | **Age≥74 years^b^** | |
|  | **Adjusted for sociodemographic factors^c^** | **Fully adjusted^d^** |  | **Adjusted for sociodemographic factors^c^** | **Fully adjusted^d^** |
| Total sedentary time | 1.51 (1.19-1.91) | 1.48 (1.11-1.98) |  | 1.23 (1.01-1.51) | 0.98 (0.76-1.26) |
| **Sedentary accumulation patterns^e^** |  |  |  |  |  |
| (1) Mean sedentary bout duration | 1.09 (1.00-1.20) | 1.07 (0.95-1.21) |  | 1.10 (1.00-1.22) | 1.02 (0.91-1.15) |
| (2) Time in prolonged (≥30 min) sedentary bouts | 1.42 (1.17-1.72) | 1.38 (1.09-1.75) |  | 1.14 (0.97-1.35) | 0.96 (0.79-1.18) |
| (3) Gini index | 1.30 (1.05-1.62) | 1.24 (1.00-1.55) |  | 0.96 (0.79-1.16) | 0.88 (0.73-1.07) |
| (4) Number of sedentary breaks | 0.74 (0.61-0.91) | 0.78 (0.63-0.97) |  | 0.92 (0.77-1.08) | 1.03 (0.86-1.23) |
| (5) Breaks per sedentary hour | 0.68 (0.55-0.85) | 0.72 (0.56-0.92) |  | 0.87 (0.73-1.05) | 1.06 (0.86-1.31) |
| (6) Alpha | 0.74 (0.60-0.91) | 0.78 (0.61-0.98) |  | 0.85 (0.71-1.01) | 0.98 (0.81-1.20) |
| Transition probability from |  |  |  |  |  |
| (7) sedentary to LIPA state | 0.72 (0.58-0.90) | 0.76 (0.60-0.96) |  | 0.90 (0.75-1.08) | 1.05 (0.86-1.29) |
| (8) sedentary to MVPA state | 0.73 (0.55-0.96) | 0.79 (0.59-1.05) |  | 0.77 (0.56-1.06) | 1.07 (0.76-1.50) |

Abbreviations: CI, confidence interval; HR, hazard ratio; PA, physical activity; LIPA, light intensity physical activity;

MVPA, moderate-to-vigorous physical activity; SD, standard deviation.

^a^N total = 2981, N events = 94, mean follow-up (SD) = 6.5 (0.5) years.

^b^N total = 965, N events = 121, mean follow-up (SD) = 6.3 (0.9) years.

^c^Models adjusted for age (time-scale), sex, ethnicity, education, occupation position, marital status and total waking day duration.

^d^Models additionally adjusted for smoking status, alcohol consumption, fruits and vegetables consumption, prevalent diabetes, BMI, hypertension, hyperlipidaemia, morbidity index and MVPA recommendation.

^e^Metrics are standardized based on sample mean & SD resulting in HRs corresponding to one SD higher value. For metrics 1-3, an increase of 1-SD corresponds to less favourable sedentary accumulation pattern. For metrics 4-8, an increase of 1-SD corresponds to more favourable sedentary accumulation pattern. 1 SD represents 100.2 minutes for total sedentary time, 6.1 minutes for mean sedentary bout duration, 143.2 minutes for time in prolonged (≥30 min) sedentary bouts, 0.036 for Gini Index, 16.0 for number of sedentary breaks, 6.2 for breaks per sedentary hour, 0.127 for Alpha, and 3.1%, and 0.5% for transition probability from sedentary to LIPA and MVPA states, respectively.

**eTable 11** Associations of total sedentary time and sedentary accumulation patterns with incident CVD and all-cause mortality adjusting for MVPA as a continuous variable in the fully adjusted model

|  | **HR (95% CI)** | | | | |
| --- | --- | --- | --- | --- | --- |
|  | **Incident CVD^a^** | |  | **All-cause mortality^b^** | |
|  | **Continuous MVPA^c^** | **Dichotomous MVPA^d^** |  | **Continuous MVPA^c^** | **Dichotomous MVPA^d^** |
| Total sedentary time | 1.06 (0.87-1.30) | 1.02 (0.88-1.19) |  | 1.09 (0.89-1.35) | 1.16 (0.98-1.38) |
| **Sedentary accumulation patterns^e^** |  |  |  |  |  |
| (1) Mean sedentary bout duration | 1.13 (1.01-1.26) | 1.09 (0.98-1.23) |  | 1.03 (0.95-1.11) | 1.03 (0.95-1.11) |
| (2) Time in prolonged (≥30 min) sedentary bouts | 1.09 (0.94-1.26) | 1.05 (0.92-1.20) |  | 1.09 (0.95-1.27) | 1.12 (0.98-1.29) |
| (3) Gini index | 1.00 (0.89-1.13) | 0.99 (0.88-1.12) |  | 1.03 (0.90-1.18) | 1.06 (0.93-1.20) |
| (4) Number of sedentary breaks | 0.92 (0.82-1.04) | 0.94 (0.84-1.07) |  | 0.92 (0.81-1.04) | 0.92 (0.81-1.05) |
| (5) Breaks per sedentary hour | 0.95 (0.82-1.09) | 0.97 (0.85-1.10) |  | 0.93 (0.80-1.08) | 0.90 (0.78-1.04) |
| (6) Alpha | 0.92 (0.80-1.05) | 0.94 (0.82-1.07) |  | 0.93 (0.81-1.07) | 0.92 (0.80-1.05) |
| Transition probability from |  |  |  |  |  |
| (7) sedentary to LIPA state | 0.96 (0.84-1.10) | 0.98 (0.86-1.11) |  | 0.94 (0.81-1.08) | 0.92 (0.80-1.06) |
| (8) sedentary to MVPA state | 0.90 (0.74-1.08) | 0.92 (0.79-1.08) |  | 0.89 (0.70-1.13) | 0.82 (0.67-1.01) |

Abbreviations: CI, confidence interval; HR, hazard ratio; PA, physical activity; LIPA, light intensity physical activity;

MVPA, moderate-to-vigorous physical activity; SD, standard deviation.

^a^N total = 3321, N events = 299, mean follow-up (SD) = 6.2 (1.3) years.

^b^N total = 3991, N events = 260, mean follow-up (SD) = 6.4 (0.8) years.

^c^Models adjusted for sociodemographic, behavioural, health-related factors and daily time in MVPA

^d^Models adjusted for sociodemographic, behavioural, health-related factors and MVPA recommendation.

^e^Metrics are standardized based on sample mean & SD resulting in HRs corresponding to one SD higher value. For metrics 1-3, an increase of 1-SD corresponds to less favourable sedentary accumulation pattern. For metrics 4-8, an increase of 1-SD corresponds to more favourable sedentary accumulation pattern. 1 SD represents 100.2 minutes for total sedentary time, 6.1 minutes for mean sedentary bout duration, 143.2 minutes for time in prolonged (≥30 min) sedentary bouts, 0.036 for Gini Index, 16.0 for number of sedentary breaks, 6.2 for breaks per sedentary hour, 0.127 for Alpha, and 3.1%, and 0.5% for transition probability from sedentary to LIPA and MVPA states, respectively.

**eTable 12** Associations of total sedentary time and sedentary accumulation patterns with all-cause mortality stratified by age adjusting for MVPA as a continuous variable in the fully adjusted model

|  | **HR (95% CI)** | | | | |
| --- | --- | --- | --- | --- | --- |
|  | **Age<74 years^a^** | |  | **Age≥74 years^b^** | |
|  | **Continuous MVPA^c^** | **Dichotomous MVPA^d^** |  | **Continuous MVPA^c^** | **Dichotomous MVPA^d^** |
| Total sedentary time | 1.44 (1.03-2.01) | 1.45 (1.12-1.88) |  | 0.89 (0.67-1.17) | 0.95 (0.76-1.20) |
| **Sedentary accumulation patterns^e^** |  |  |  |  |  |
| (1) Mean sedentary bout duration | 1.06 (0.96-1.18) | 1.06 (0.96-1.18) |  | 1.00 (0.89-1.12) | 1.00 (0.89-1.12) |
| (2) Time in prolonged (≥30 min) sedentary bouts | 1.36 (1.08-1.70) | 1.39 (1.13-1.71) |  | 0.94 (0.77-1.14) | 0.96 (0.80-1.15) |
| (3) Gini index | 1.26 (1.02-1.54) | 1.30 (1.06-1.58) |  | 0.90 (0.75-1.08) | 0.92 (0.77-1.09) |
| (4) Number of sedentary breaks | 0.77 (0.64-0.93) | 0.77 (0.63-0.94) |  | 1.03 (0.88-1.22) | 1.04 (0.89-1.23) |
| (5) Breaks per sedentary hour | 0.75 (0.60-0.95) | 0.73 (0.59-0.91) |  | 1.09 (0.90-1.33) | 1.08 (0.89-1.30) |
| (6) Alpha | 0.82 (0.66-1.02) | 0.80 (0.65-0.98) |  | 1.03 (0.86-1.24) | 1.02 (0.85-1.23) |
| Transition probability from |  |  |  |  |  |
| (7) sedentary to LIPA state | 0.78 (0.62-0.97) | 0.75 (0.61-0.94) |  | 1.09 (0.90-1.31) | 1.08 (0.89-1.30) |
| (8) sedentary to MVPA state | 0.85 (0.62-1.16) | 0.77 (0.59-1.00) |  | 0.99 (0.68-1.46) | 1.08 (0.90-1.29) |

Abbreviations: CI, confidence interval; HR, hazard ratio; PA, physical activity; LIPA, light intensity physical activity;

MVPA, moderate-to-vigorous physical activity; SD, standard deviation.

^a^N total = 3001, N events = 114.

^b^N total = 990, N events = 146.

^c^Models adjusted for sociodemographic, behavioural, health-related factors and daily time in MVPA.

^d^Models adjusted for sociodemographic, behavioural, health-related factors and MVPA recommendation.

^e^Metrics are standardized based on sample mean & SD resulting in HRs corresponding to one SD higher value. For metrics 1-3, an increase of 1-SD corresponds to less favourable sedentary accumulation pattern. For metrics 4-8, an increase of 1-SD corresponds to more favourable sedentary accumulation pattern. 1 SD represents 100.2 minutes for total sedentary time, 6.1 minutes for mean sedentary bout duration, 143.2 minutes for time in prolonged (≥30 min) sedentary bouts, 0.036 for Gini Index, 16.0 for number of sedentary breaks, 6.2 for breaks per sedentary hour, 0.127 for Alpha, and 3.1%, and 0.5% for transition probability from sedentary to LIPA and MVPA states, respectively.

**SUPPLEMENTARY FIGURES**

**eFigure 1** Participant flow chart


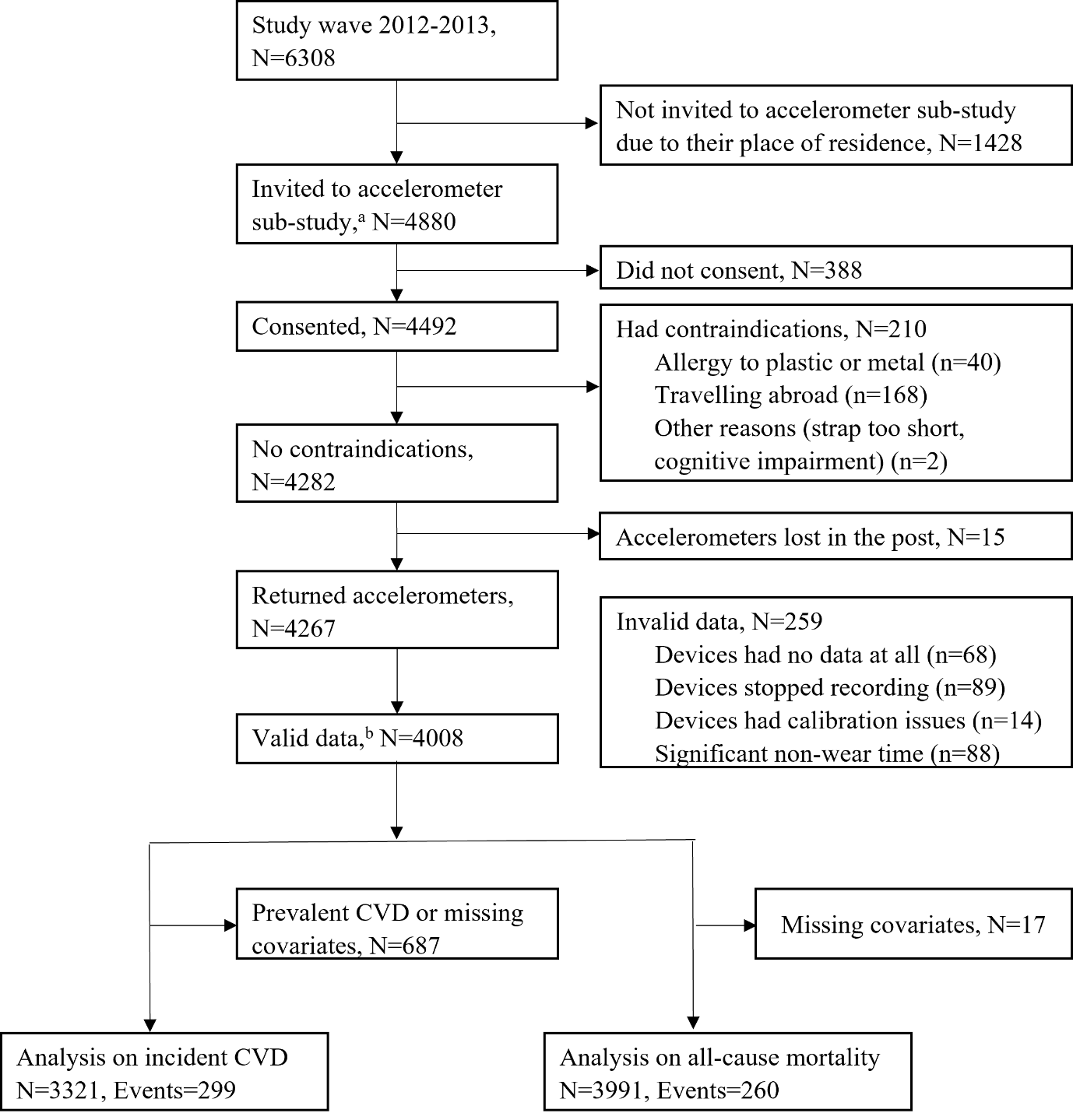


^a^4680 seen at the London clinic and 200 at home.

^b^Defined as daily wear time ≥2/3 of waking hours, for at least 2 weekdays and 2 week-end days. In total, 97.6% of 3991 participants (largest analytical sample) had valid data for 7 days, 1.3% for 6 days, 0.6% for 5 days, and 0.5% for 4 days.

**eFigure 2** Associations of total sedentary time and sedentary accumulation patterns with all-cause mortality stratified by median age of the sample population


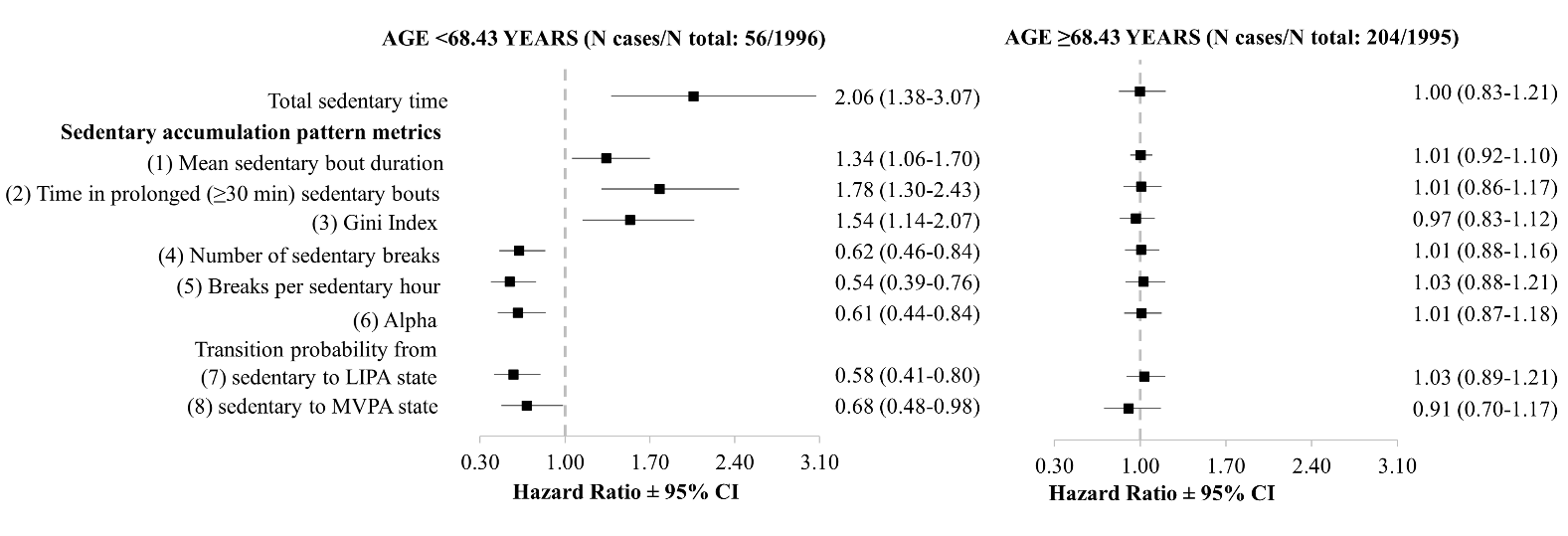


Abbreviations: PA, physical activity; LIPA, light intensity physical activity; MVPA, moderate-to-vigorous physical activity.

Models adjusted for age (as timescale), sociodemographic, behavioural, health-related risk factors and MVPA recommendation. Metrics are standardized based on sample mean & SD resulting in HRs corresponding to one SD higher value. For metrics 1-3, an increase of 1-SD corresponds to less favourable sedentary accumulation pattern. For metrics 4-8, an increase of 1-SD corresponds to more favourable sedentary accumulation pattern. 1 SD represents 100.2 minutes for total sedentary time, 6.1 minutes for mean sedentary bout duration, 143.2 minutes for time in prolonged (≥30 min) sedentary bouts, 0.036 for Gini Index, 16.0 for number of sedentary breaks, 6.2 for breaks per sedentary hour, 0.127 for Alpha, and 3.1%, and 0.5% for transition probability from sedentary to LIPA and MVPA states, respectively.
